# How do depressed patients evaluate their quality of life? A qualitative study

**DOI:** 10.1186/s41687-018-0076-z

**Published:** 2018-11-05

**Authors:** Jacques J. Zimmermann, Maria Lucia Tiellet Nunes, Marcelo P. Fleck

**Affiliations:** 1 Postgraduate Program in Psychiatry and Behavioral Sciences, Universidade Federal do Rio Grande do Sul, Porto Alegre, Brazil; 2 Pontifícia Universidade Católica do Rio Grande do Sul, Faculty of Psychology, Porto Alegre, Brazil; 3 Department of Psychiatry and Legal Medicine, Universidade Federal do Rio Grande do Sul, Porto Alegre, Brazil

**Keywords:** Quality of life, Depression, Qualitative study, Whoqol-bref

## Abstract

**Background:**

This is a qualitative study that aims to investigate the effect of depressive status on responses to items on the WHO quality of life assessment instrument (WHOQOL-Bref), comparing which aspects of the individual’s life he or she takes into account in responding to the items related to quality of life when depressed and when euthymic.

**Results:**

Six adult women were interviewed prior to initiating treatment for a depressive episode and were then interviewed again six months later when in remission from the episode. The ‘think aloud’ method of cognitive interviewing was used. Based on the Wilcoxon test, the seven items that exhibited a significant change with the improvement of the depressive condition were examined in depth, and the think aloud method was used to reveal the subjects’ cognitive processes. Depressive symptoms were associated with different interpretations of some items and the response scale. Also, for some items, patients chose the same response for the item both times but justified their choice differently during the depressive episode and in euthymia.

**Conclusions:**

We found that, in addition to the impact caused by depression on quality of life, there are peculiarities in the way the depressed individual makes subjective assessments. We believe that qualitative studies such as the present one may provide important support in the interpretation of quantitative results.

**Electronic supplementary material:**

The online version of this article (10.1186/s41687-018-0076-z) contains supplementary material, which is available to authorized users.

## Background

The concept of quality of life is centered on the individual’s perception of his health status and his position in the sociocultural context in which he lives and in relation to his expectations and to other aspects of his life that he values [14]. As quality of life measurement instruments have become available, these have become tools used to evaluate the subjective perception of the effect that disease produces on a subject’s quality of life. Depression is expected to negatively impact quality of life because it alters the way one perceives oneself, the world, and the future (Beck’s cognitive triad; [3]). Depression has been found to have a significantly greater negative association with quality of life than other chronic medical conditions [6]. For example, patients with major depression experience significantly worse quality of life when compared with individuals with extremely debilitating physical illness, such as end-stage renal disease [5].

From a theoretical point of view, Berlim and Fleck [4] proposed that overlaps in the measurement of depression and quality of life can occur at three levels: (a) conceptual, in which depression and quality of life are representations of the same phenomenon and are thus tautological measures; (b) mediator, in which the depressive state leads to distorted (negative) perceptions of the domains that make up quality of life; and (c) metrics, as although depression and quality of life are different constructs, many items that make up a quality of life measure are also used to evaluate depression. Based on the notion of a mediating variable relationship, they postulated that negative visions of the self and the world along with hopelessness, as described by Beck et al. [3], could influence the perception of reality. It is expected that a depressed individual will evaluate the different domains of his/her life in a negative way and that, after remission, he/she will evaluate the same reality in a different and more positive way.

The present study aims to investigate the effects of the depressive status on responses to the items on the WHO quality of life assessment instrument (WHOQOL-Bref instrument), specifically by comparing which aspects of life subjects take into account when responding to items related to quality of life when depressed and when euthymic in order to verify if the quality of life of a depressed patient is indeed markedly impacted by depression or if the observed worsening is a result of distortions in the depressed patient’s subjective assessments.

The objectives of this study were to verify which items of a quality of life instrument varied significantly after remission of the depressive symptoms and to compare the justifications and experiences evoked by the patients to explain their responses during the depressive episode and after achieving euthymia.

## Methods

The qualitative method of cognitive interviewing was used [16]. Specifically, the interviewer read the items in the Brazilian WHOQOL-Bref instrument [10], and the respondent ‘thought aloud’ to provide information on their cognitive process in choosing their answer to each of the 26 items in the instrument in the original order. The qualitative method of cognitive interviewing allows a limited number of research subjects. Based on the concept of saturation in qualitative research, for the purposes of this study, we established five as the number of research subjects. We chose to examine a homogeneous group to attempt to control other intervening variables. The group chosen represented the typical patient seeking outpatient care: the adult depressed woman.

Seven adult women were interviewed at the time of their evaluation prior to initiating treatment for a unipolar depressive episode at a research outpatient clinic; six were interviewed again 6 months later when they were expected to be in remission. Patients with bipolar disorder or psychiatric or clinical comorbidities were excluded from the study. The study inclusion criteria were female gender, age between 18 and 60 years old, good ability to express and narrate experiences, a Beck Depression Inventory score [8] greater than nineteen points in the first interview and less than ten points in the second, indicating remission of the depressive symptoms, and agreement to participate and willingness to undergo a second interview 6 months later. The interviews were recorded and fully transcribed. There was the possibility of discarding some idiosyncratic private interview, which did not occur in this sample. Two meetings took place. After signing informed consent forms, the subjects completed the BDI, and the first cognitive interview was held. Six months later, we scheduled a meeting in which the BDI was again completed, and the second cognitive interview was then conducted. All of the patients received selective serotonin reuptake inhibitors.

Subsequently, a quantitative methodology was used to select the items whose scores changed significantly between the first interview and the second one, which was performed 6 months later. The Wilcoxon test, a nonparametric statistic test, was used to compare the WHOQOL-Bref scores between the time points.

The Project was approved by the research ethics committees at the Clinics Hospital of Porto Alegre and the São Pedro Psychiatric Hospital. The informed consent forms were approved by the research committees and were presented to the subjects before their participation in the study. Confidentiality of the materials was respected.

## Results

All seven invited patients met the inclusion criteria. Two of the seven interviewees abandoned treatment at the outpatient clinic. Of these, one could not be found, but the other one was located after the six-month period, had been treated at another service with a decrease in depressive symptoms, and responded to the second interview. Thus, six interviewees experienced remission of the depressive episode and were included in the study, totaling twelve interviews. Demographic data are presented in Table 1. The treatment used was the same for all, as all of the subjects presented with unipolar depression without other comorbidities. The interviews lasted approximately 30 minutes during the depressive episode and 1 hour when in euthymia.

**Table 1:** Characteristics of the participants

Patient	Age	Marital status	Occupation’	BDI (T1)	BDI (T2)
1	37	single	radiology technician	33	4
2	45	married	seam stress	28	8
3	44	widow	cleaning lady	28	9
4	25	single	student	23	1
5	48	married	cook	38	7
6	55	divorced	saleswoman’	31	8

The seven items of the WHOQOL-bref that exhibited a significant change (*p* < 0.05) in scores from the first to the second interview are examined in depth below, namely, item 1, quality of life perception; four physical domain items, including item 3, pain and discomfort, item 4, medication or treatment dependence, item 16, sleep and rest, and item 18, working capacity; and two items from the psychological domain – item 6, spirituality/religion/personal beliefs and item 7, thinking, learning, memory and concentration (Additional file 1). As this is a qualitative study, sections of the patients’ verbal responses will be used to illustrate the reports.

### Item 1 – Overall quality of life. How would you rate your quality of life?

In the first interview, five of the six interviewees reported that the depressive symptoms interfered with their quality of life, which showed a clear change in the way they perceived common events.

Interviewee 2: *“I do not feel good inside my house, right? I have no pleasure in having a good diet, in making food for myself and my family. I have no motivation to do my tasks, right? And ... I’ve cried a lot, I’ve been very emotional.”*

Interviewee 4: *“Yeah, I got away from everybody. Before I was not like this, I did not move away, I did things ... When I was fine, I had more pleasure in doing things, and in meeting people.”*

Only one interviewee classified her quality of life as good in the first interview, focusing on objective aspects of reality:

Interviewee 6: *“I live well, I eat well ... I have opportunities to go out, although I do not want to.”*

In the second interview, the same interviewee maintained the same response because she changed the evaluation criterion used: in the first interview, she used a more objective criterion of reality, as if she was evaluating her ‘standard of living,’ but in the second interview, she took into account the emotional problems faced as a result of the depressive episode, comparing the two instances, recognizing how much she had improved with the treatment:

Interviewee 6: *“... I am living a very quiet life. I was a person very concerned about my children. I can now separate and don’t have that concern of [knowing] where they are.” “I was very worried that something would happen, violent robbery ... an accident. And today, about quality of life, I feel calm about it.”*

In the first interview, in the presence of severe depressive symptoms, four of the six interviewees evaluated their quality of life as “neither bad nor good;” the qualitative examination of these answers leads to an interesting speculation in this case: the neither bad nor good answer seems to have been considered worse than bad or very bad because it denotes indifference. At this stage, neither bad nor good does not represent the midpoint of the Likert scale but has another connotation, apathy, expressed by one of the interviewees through a Portuguese idiom:

Interviewee 5: *“Whatever.” [I couldn’t care less.]*

Interviewee 1: *“I’m inert, do not have as much taste, neither bad, nor good.”*

The Likert scale of the WHOQOL-Bref has an odd number of responses with a midpoint that usually expresses a ‘neutral’ position of the responder because the Likert scale is bipolar, ranging from one end to the other between two opposing constructs [7].

In the second interview, naturally, a greater diversity of responses appears due to the improvement of the depressive symptoms, when the personal characteristics of the interviewees emerge, while the depressive symptoms seemed to homogenize the sample in the first interview. At this second point, five of the six interviewees evaluated their quality of life as “good,” and one evaluated it as “very good.” Only one interviewee considered the situation experienced during the depressive episode as comparable to the situation of euthymia. The others only made an evaluation recognizing aspects that they could not perceive in the presence of the depressive symptoms such as material possessions, acquisitions, achievements, and ability to work, which came to have a greater value when the depressive symptoms subsided.

### Item 3 – Physical domain: Pain and discomfort. To what extent do you think your (physical) pain prevents you from doing what you need?

This is a facet that explores unpleasant physical sensations experienced by the individual, especially pain. Two interviewees, even though they did not suffer physical pain, answered “extremely” in the first interview, attributing a somatic connotation to the depressive experience:

Interviewee 1: *“Because ‘it’ [the depression] has ‘caused’ me an effort that before I did not ... need to make.”*

Interviewed 2: *“Physical pain... It would not be a physical pain, but I feel like sleeping all the time. Very discouraged! It would not be physical pain, but extreme discouragement!”*

A third answered “more or less,” although she did not clearly present physical pain and her clinician had attributed her somatic symptoms to her depressive state.

Interviewee 3: *“I don’t know... Well... Digestive problems, stomach problems... Even because of depression too, right? It generates fear like that, and then, when I already have the problem of pain, it prevents me from going out. It messes me up a lot.” “Yeah, then the doctor sent me to a specialist. Because... She thinks that my pains have to do with depression. If I treat, all of a sudden, I get better, right?”*

Two interviewees reported having very little pain, while the sixth interviewee did actually suffer from physical pain.

Depression is associated with physical symptoms, and improvements in these symptoms are correlated with improvements to depressive symptoms, suggesting that a patient’s ability to obtain remission from depression may be directly related to the reduction in physical symptoms [15]. Patients’ verbal responses suggest that the symptoms of depression can be interpreted as physical pain, which may be a way of showing emotional distress.

The two respondents who answered “extremely” when questioned about physical pain in the first interview answered “more or less” in the second interview: one was in a postoperative period, and the other claimed pain in the body. The four others answered “nothing,” although they eventually mentioned pain, showing that they understood correctly the meaning of the item because the physical pain did not prevent them from doing what they needed.

This item of the instrument was interpreted differently than expected at the time of the depressive episode, presenting significant change only due to the overestimation of the item in the first interview.

The relationship between perceptions of physical pain and depression is complex and inter-related at a clinical level [13], and there are probably many commonalities between them from a neurobiological perspective [11].

### Item 4 – Physical domain: Dependence on medication or treatments. How much do you need medical treatment to maintain your daily life?

In the first interview, four respondents considered that they really needed medical treatment, even though treatment had not yet started.

Interviewee 1: *“If it is mental, ‘extremely,’ … because I tried by myself and couldn’t.”*

Interviewee 2: *“Because I feel the need to have a medical follow-up, I spent a lot of time with depression without seeking medical help, and now I’m on the edge.”* About the first dose of antidepressant medication: *“It already had an effect! I felt strong relief already with the first dose.”*

One interviewee, despite having answered “extremely,” attributed her illness to somatic problems, which in her evaluation were the cause of her suffering.

Interviewee 6: *“Because I’m feeling sick... I feel back pain, I feel a headache, I feel pain in my feet, my feet burn a lot.”*

Another compared herself with other people and thought she was not sick enough to consult doctors.

Interviewee 3: *“Comparing myself to other people, I think I do not always have to be at the doctor’s office. Do you understand? Because I’m not such a sick person to always be at the doctor’s office. So, I think it’s little. Very little, right?”*

Although they were in a specialized outpatient clinic, these two interviewees did not take into account the depressive episode in justifying their responses.

In the second interview, 6 months after the beginning of the treatment, the women interviewed continued to have consultations every 2 weeks and receive antidepressant medication. There was, however, a tendency to refuse to acknowledge the importance of treating depression in the substantial improvement they had achieved. Two answered “nothing” and four “more or less” to the question about dependence on medication or treatments.

Thus, although before the beginning of the treatment they expressed positive expectations in relation to it, after therapeutic success, there was no recognition of the medical treatment as having been the agent of the improvement presented. A first hypothesis is a tendency not to consider the treatment of depression as a medical treatment.

Interviewee 3: *“Only the antidepressant, at the moment I’m doing the treatment.”* The interviewer asks again how much she needed some medical treatment to get through life: *“Nothing, right?”*

Interviewee 5: *“No, I don’t need any medical treatment.” “No, nothing! Except here, right? Outside of here nothing.” “These degenerative diseases, right, you are dependent on a doctor. If you have not taken medicine, you’re not okay ... So, I don’t need a doctor like that...”*

Interviewee 6: *“Ah, doctor ... Not enough and not extremely, but I know I need it.” “It’s more than ‘more or less’ and less than ‘enough’.”*

It is noteworthy that the interviewees do not attribute the improvement of the symptoms to the medication treatment. This could explain the high rates of dropout from depression treatment observed after clinical improvement, which at 4 weeks reaches between 29% and 42%, increasing at 6 months of treatment to 63 to 76% [12].

### Item 16 – Physical domain: Sleep and rest. How satisfied are you with your sleep?

This facet relates sleep and rest to quality of life. There is a complex relationship between sleep and depression, but sleep disturbances are among the diagnostic criteria for major depressive disorder according to the DSM-5 [9], which can be seen in the first interviews.

Interviewee 1: *“Because at one point I wasn’t able to sleep, and now, in this last period, I’m sleeping too much.”*

Interviewee 2: *“I cannot sleep properly; I always wake up at night. And I wake up very tired. I don’t have a restful sleep.”*

Interviewee 3: *“I don’t sleep like I used to, that heavy sleep, no! Never anymore! And I wake up easily with any little noise. And then, falling asleep is a sacrifice.”*

Another point to highlight is that the “neither bad nor good” response of one of the interviewees in the first interview had an indifference connotation, as occurred in item 1 of the instrument:

Interviewee 5: *“There are days when I sleep too much. Other days, I sleep less. So I think ‘neither more neither less’ [neither bad nor good].”*

When the interviewer remarked on the interviewee’s consideration that, because she was not working, her severe sleep difficulties did not seem to be a problem at the time, she explained the indifference of her choice with an idiosyncratic interpretation, ‘averaging’ the days of insomnia and hypersomnia:

Interviewee 5: *“No, because if I had to get up the other day, at a fixed time, it would be a lot harder, right?”*

In the second interview, three interviewees presented significant improvements regarding sleep satisfaction, two presented partial improvement, and one kept her answer unchanged.

Interviewee 1: “*In the old days, when I lay down, I thought a thousand things before going to bed. And the thoughts, they continued, and ‘gave origin to each other.’ And now, no, I lie down and sleep!”*

Interviewee 4: *“Oh, it’s just that previously I couldn’t sleep. It’s just that I spent a good amount of time sleeping only four hours. Sometimes I was not sleepy, I kept rolling and rolling on the bed and not sleeping. There were days when I slept too much... Then, I slept and could not wake up.” “But now it’s normal! At least it’s good for me, I sleep nearly seven hours.”*

### Item 18 – Physical domain: Working capacity. How satisfied are you with your ability to work?

This is another item very related to depressive symptoms. Depression is known to have a greater negative impact on labor performance than other diseases such as rheumatoid arthritis, asthma, lower back pain, headache, hypertension, arthritis and allergies [1]. One interviewee reported being “very dissatisfied,” three were “dissatisfied,” and one was “neither satisfied nor dissatisfied;” one interviewee did not respond.

Interviewee 1: *“I am dissatisfied, but I can do what I must do, but with great effort.”*

Interviewee 2: *“I know I can do more. And... And I’m not succeeding.”*

Interviewee 3: *“I’ve been more capable, now it seems I’m getting more and more discouraged.”*

Two interviewees were unemployed at the time of the first interview. One of them evaluated that she was “neither satisfied nor dissatisfied” because she was not using her ability to work at that moment.

Interviewee 4: *“Ah, when I work, I work hard, right? And everyone always praises me, right? But I think this has diminished now that I’m more tired, right? I was not so much; a while ago I had not so much... I had more willingness! So I guess it’s more or less...”*

All the interviewees evaluated improved in the second interview (there was one missing response). There was a significant improvement in two interviewees and a slight improvement in the other three.

Interviewee 5: *“Oh, I think of my role, what I do, right? And in my creativity. I don’t know ... And I’m not lazy in terms of studying, seeking out knowledge, looking for more information. So, with regard to work, I am very satisfied.”*

One interviewee presented only a slight improvement due to a momentary situation: a surgical postoperative condition that caused physical restrictions that made her question her ability to work.

### Item 6 – Psychological domain: Spirituality/religion/personal beliefs. To what extent do you think your life makes sense?

This was an item that did not pose difficulties with regard to the answer, showing a very homogenous behavior related to the depressive episode. Five of the six interviewees answered “nothing” and “more or less” in the first interview, expressing significant impairment caused by the depressive episode.

Interviewee 1: *“Because I don’t see... (Cries). I see no point. It seems like everything I had to do, I already did.”*

Interviewee 2: *“It’s because I have my 10-year-old son, right? And, to me, the meaning is him.” “Yeah, it is my youngest son! (Cries a lot) That he still needs me a lot.”*

Interviewee 3: *“The daughters! They give me much strength to move on, to continue life, right? If it were not for them, I don’t even know if I would be here today.”*

Interviewee 5: *“Because the children are already raised, right?” “I cannot explain. Nothing. There’s nothing!” “It’s just a hole! Just a hole...”*

Interviewee 6: *“Now the meaning of my life is only my children.” “Because that’s what’s left, right? Of anything good.”*

Only one interviewee, a practitioner of Kardecist Spiritism, estimated that her life made perfect sense already in the first interview. The religious coping strategies of Kardecist Spiritism seem to have neutralized, at that moment, the impact of the depressive episode.

Interviewee 4: *“If you’re here, you can’t be kidding. I think we’re here to, I don’t know, to live and help people, like this.”*

In the second interview, this same interviewee improved her evaluation, responding “extremely,” again providing a spiritual explanation.

Interviewee 4: *“I’m a spiritist, right? So I believe we reincarnate and things like that. So I think we come like this, we’re not here for nothing. There’s a reason!”*

All the interviewees improved their evaluation of this item in the second interview:

Interviewee 2: *“Ah, because it’s a blessing to be... alive and have the opportunity to live and do the things that we have the capacity to do, right? That’s it!” “I’m sure that even the fact I came to work... Come here to work on my neurological side, it was like this, for me, it was God who sent me here. Because I was like... Deep down, right?”*

Interviewee 3: *“Life is important, right? I think life is important. In spite of the problems that people go through, and the depression, which takes all the pleasure of our everyday life, life is important.”*

Interviewee 5: *“Oh, I’m very much needed by some people. So, it is extremely. Making sense is when someone misses me. It’s that.”*

Interviewee 6: *“...I think my children are healthy, no one does drugs, no one smokes. They are all happy, all studying, working... It is the parameter that guides the satisfaction of my life.”*

Four of them related the meaning of life to their offspring. Children and religion appear as a protective factor in the first interview and as a source of satisfaction in the second.

### Item 7 – Psychological domain: Thinking, learning, memory and concentration. How much can you focus on? Nothing, very little, more or less, enough or extremely?

In the first interview one patient answered “nothing,” three “very little,” and two “more or less.” There was a significant improvement in the interviewees’ assessment of their ability to concentrate after 6 months of treatment.

Interviewee 1: *“Because lately, sometimes I get a book to read, and I give up the book. I look at the TV, but I’m not looking at the TV either. And then I listen to the radio, but I’m not listening to the radio either.”*

Interviewee 2: *“I get restless! I sit. In my job... I sew. And I have that urge to sit, I have an obligation to be sitting there, doing, and at the same time, I have the desire to leave.”*

Interviewee 4: *“There, in doing some activity, task or paying attention to something, I cannot stay very long. Soon I’ll be ... I don’t know, thinking of something else, or something that passes by catches my attention.”*

Interviewee 5: *“And I watch a movie, so, if I get to the end, sometimes I do not even know what happened, I cannot even... They comment, right, and I cannot follow the reasoning.”*

Interviewee 6: *“Ah, if I read a book, I have to read the same page two or three times. And I cannot stand sitting like this. It’s getting difficult to stay here, like this, listening...”*

In the second interview, there was improvement of the evaluation of five interviewees:

Interviewee 1: *“I’m in a ‘Zen’ period, right? Because I’m on vacation, so, everything I’m doing is just what I feel like doing (laugh a lot). So my... I’m focusing my thoughts on what I’m doing.”*

Interviewee 4: *“Oh, my concentration improved a lot... Look, I think that it was a lot, not extremely, but the last few times I had to concentrate I got it!” “I could not concentrate to get things done because I was graduating, finishing my course work. So those last few months, I could concentrate more, do the activities and finish.”*

Only one interviewee maintained her evaluation from the first interview 6 months later, responding “very little” in the two interviews. Due to the improvement of her symptoms, she had changed jobs, so that in the second interview she was facing a new challenge that distressed her. She responded in the same way in both instances, although they were very different external situations because the improvement of her depressive symptoms allowed her to start a new work activity. It was possible to observe that concentration capacity, in this case, was related to demands. The improvement of the depressive symptoms caused this woman to seek more complex activities, which demanded greater concentration, so that we evaluated the interviewee in the face of very diverse life situations in this interval of 6 months.

## Discussion

The scores of WHOQOL-Bref items 6, spirituality, religion, and personal beliefs, 7, thinking, learning, memory, and concentration and 18, working capacity, changed significantly between the first and second interviews, as expected, because they are items that are naturally very related to depression.

WHOQOL-Bref item 1, global quality of life, presented a specific problem in its functioning in this group of depressed patients. While the initial estimate was indeed overvalued because the “neither bad nor good” response was considered worse than the “bad” and “very bad” alternatives, there was an underestimation of the improvement obtained following the treatment of the depressive episode in the second interview. Nevertheless, the change in the scores for this item was significant according to the Wilcoxon test, which indicates that it is an item so strongly related to depression that it resisted a presumed systematic error of interpretation during the depressive episode.

There was also a problem in the functioning of item 3, investigating pain and discomfort, but only during the depressive episode, due to the lack of discernment of physical pain and emotional suffering verified in these depressed patients. After a decline of the symptoms, the interviewees had no difficulty distinguishing between physical and emotional suffering.

Finally, there was an unexpected significant reduction in the score of item 4, dependence of medication or treatments. In theory, there should have been no reason for such dependence to change as the depressive symptoms subsided because the effectiveness of a treatment does not diminish the need for it. Despite documented efficacy, a high proportion of patients with mental disorders withdraw from treatment, and unfortunately, the perception of little need for treatment is a major barrier to seeking and maintaining treatment for mental disorders worldwide [2].

The present study has some limitations. Because it is a qualitative study, the data are from a small, non-representative sample. The fact that the sample only includes women restricts the range of its results. However, the results from the qualitative methodology generate a hypothesis that reveals phenomena that are hidden when only examined through a quantitative approach. Despite the small sample size, the Wilcoxon signed rank test was used to perform an in-depth examination of the items that varied significantly between the two time points, as these were the items with a higher probability of presenting with differences in qualitative aspects among the interviewees. It was not the objective of the present study to use a quantitative methodology to evaluate responses to treatment but rather to identify items more sensitive to change in the study sample and, therefore, those with a greater probability of presenting with specific qualitative characteristics.

The application of the qualitative method of cognitive interviewing was an attempt to reveal, in addition to the scores of the scales, what individuals take into account in answering the items of the instrument. We verified that, in addition to the impact of depression on quality of life, there are peculiarities in the way depressed individuals make subjective assessments. Understanding the biases caused by a mental condition can help improve the items of the instrument and choose more appropriate instruments, as it deepens the understanding of why some instruments work better for certain pathologies. We believe that qualitative studies such as the present may provide important help in the interpretation of quantitative results.

## Additional file

Additional file 1: Wilcoxon Signed Ranks Test Table. (DOCX 14 kb)
