# Supplementary material for: How do depressed patients evaluate their quality of life? A qualitative study
Source: J Patient Rep Outcomes. 2018 Nov 5;2:52. doi: 10.1186/s41687-018-0076-z (PMC6215789; doi:10.1186/s41687-018-0076-z)
Supplement: Supplementary file 1 — Wilcoxon Signed Ranks Test Table. (DOCX 14 kb) [file 41687_2018_76_MOESM1_ESM.docx]

| **Wilcoxon Signed Ranks Test** | | |
| --- | --- | --- |
| Item | Z | Asymp. Sig. (2-tailed) |
| 1 | -2.070^a^ | .038 |
| 2 | -1.826^a^ | .068 |
| 3 | -2.271^b^ | .023 |
| 4 | -2.232^b^ | .026 |
| 5 | -1.841^a^ | .066 |
| 6 | -2.226^a^ | .026 |
| 7 | -2.060^a^ | .039 |
| 8 | -1.807^a^ | .071 |
| 9 | -1.134^a^ | .257 |
| 10 | -1.633^a^ | .102 |
| 11 | .000^c^ | 1.000 |
| 12 | -1.633^a^ | .102 |
| 13 | -1.342^a^ | .180 |
| 14 | .000^c^ | 1.000 |
| 15 | -1.890^a^ | .059 |
| 16 | -2.041^a^ | .041 |
| 17 | -1.160^a^ | .246 |
| 18 | -2.226^a^ | .026 |
| 19 | -1.826^a^ | .068 |
| 20 | -1.473^a^ | .141 |
| 21 | -1.414^a^ | .157 |
| 22 | -1.511^a^ | .131 |
| 23 | -1.890^a^ | .059 |
| 24 | -1.000^a^ | .317 |
| 25 | .000^c^ | 1.000 |
| 26 | -1.633^b^ | .102 |

a. Based on negative ranks.

b. Based on positive ranks.

c. The sum of negative ranks equals the sum of positive ranks.
